# (*E*)-1-(4-Bromo­phen­yl)-3-(3,4,5-trimethoxy­phen­yl)prop-2-en-1-one^1^

**DOI:** 10.1107/S1600536808041780

**Published:** 2008-12-13

**Authors:** Thitipone Suwunwong, Suchada Chantrapromma, Hoong-Kun Fun

**Affiliations:** a Crystal Materials Research Unit, Department of Chemistry, Faculty of Science, Prince of Songkla University, Hat-Yai, Songkhla 90112, Thailand; b X-ray Crystallography Unit, School of Physics, Universiti Sains Malaysia, 11800 USM, Penang, Malaysia

## Abstract

In the title compound, C_18_H_17_BrO_4_, the dihedral angle between the 4-bromo­phenyl and 3,4,5-trimethoxy­phenyl rings is 44.18 (6)°. In the crystal structure, the mol­ecules are linked by C—H⋯O and C—H⋯π inter­actions.

## Related literature

For background and applications to chalcones, see: Jung *et al.* (2008 ▶); Patil *et al.* (2007 ▶); Patil & Dharmaprakash (2008 ▶); Prasad *et al.* (2008 ▶); Schlogl & Egger (1963 ▶). For related structures, see: Ng *et al.* (2006 ▶); Patil *et al.* (2006 ▶; 2007 ▶). For on hydrogen-bond motifs, see: Bernstein *et al.* (1995 ▶). For bond-length data, see: Allen *et al.* (1987 ▶).
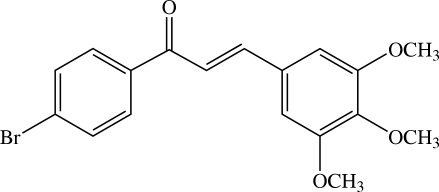


## Experimental

### 

#### Crystal data

C_18_H_17_BrO_4_
*M* *_r_* = 377.22
Tetragonal, 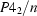

*a* = 26.6517 (3) Å
*c* = 4.4238 (1) Å
*V* = 3142.28 (9) Å^3^
*Z* = 8
Mo *K*α radiation
μ = 2.63 mm^−1^
*T* = 100.0 (1) K
0.55 × 0.12 × 0.12 mm

#### Data collection

Bruker SMART APEXII CCD area-detector diffractometer
Absorption correction: multi-scan (*SADABS*; Bruker, 2005 ▶) *T*_min_ = 0.320, *T*_max_ = 0.726
142737 measured reflections
9693 independent reflections
6638 reflections with *I* > 2σ(*I*)
*R*_int_ = 0.062

#### Refinement

*R*[*F* ^2^ > 2σ(*F* ^2^)] = 0.033
*wR*(*F* ^2^) = 0.097
*S* = 1.07
9693 reflections
211 parameters
H-atom parameters constrained
Δρ_max_ = 0.71 e Å^−3^
Δρ_min_ = −0.56 e Å^−3^

### 

Data collection: *APEX2* (Bruker, 2005 ▶); cell refinement: *SAINT* (Bruker, 2005 ▶); data reduction: *SAINT*; program(s) used to solve structure: *SHELXTL* (Sheldrick, 2008 ▶); program(s) used to refine structure: *SHELXTL*; molecular graphics: *SHELXTL*; software used to prepare material for publication: *SHELXTL* and *PLATON* (Spek, 2003 ▶).

## Supplementary Material

Crystal structure: contains datablocks global, I. DOI: 10.1107/S1600536808041780/pk2134sup1.cif

Structure factors: contains datablocks I. DOI: 10.1107/S1600536808041780/pk2134Isup2.hkl

Additional supplementary materials:  crystallographic information; 3D view; checkCIF report

## Figures and Tables

**Table 1:** Hydrogen-bond geometry (Å, °)

*D*—H⋯*A*	*D*—H	H⋯*A*	*D*⋯*A*	*D*—H⋯*A*
C11—H11*A*⋯O1^i^	0.93	2.52	3.4391 (16)	170
C17—H17*C*⋯O3^ii^	0.96	2.52	3.2789 (16)	136
C16—H16*B*⋯*Cg*1^iii^	0.96	2.97	3.8080 (14)	147

Symmetry codes: (i) 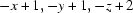; (ii) 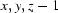; (iii) 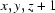. *Cg*1 is the centroid of the C10–C15 ring.

**Fig. 1.**
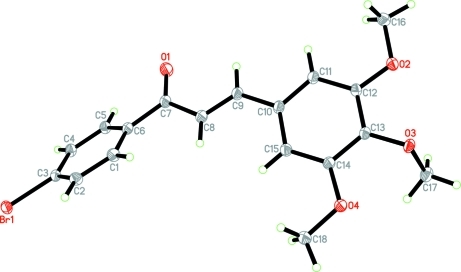
The molecular structure of the title compound, showing 50% probability displacement ellipsoids and the atom-numbering scheme.

**Fig. 2.**
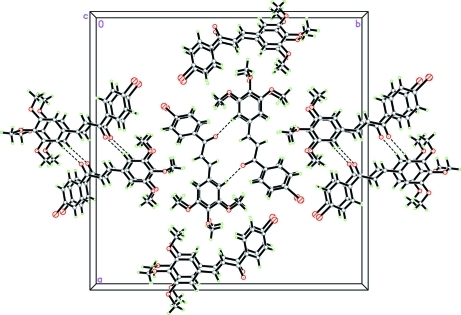
The crystal packing of the title compound, showing dimers stacked along the c axis. Hydrogen bonds are shown as dashed lines.

## supplementary crystallographic information

### Comment

Chalcones are compounds in a family of aromatic ketones with two aromatic groups bridged by an enone linkage (Ar-COCH=CH—Ar) (Schlogl & Egger, 1963). They have a wide range of applications covering non-linear optical (NLO) (Patil & Dharmaprakash, 2008) and electro-active fluorescent materials (Jung *et al.*, 2008) to materials with various biological activities. As an example, 1-(4-hydroxyphenyl)-3-(3,4,5-trimethoxyphenyl)-propenone was found to be able to inhibit growth of some bacteria (Prasad *et al.*, 2008). These interesting properties of chalcones led us to synthesize the title compound so as to study for its antibacterial and cytotoxic activities.

The molecule of the title chalcone derivative (Fig. 1) exists in an *E* configuration with respect to the C8═C9 double bond [1.3428 (17) Å] with torsion angle C7–C8–C9–C10 = -173.04 (12)°. The whole molecule is not planar as the interplanar angle between 4-bromophenyl and 3,4,5-trimethoxyphenyl rings is 44.18 (6)°. The propenone unit (C7—C9/O1) is nearly planar with the torsion angle O1–C7–C8–C9 = 3.4 (2)°. Atoms O1, C6, C7, C8 and C9 lie on the same plane with the most deviation of -0.018 (1) Å for atom C8. The mean plane through O1/C6/C7/C8/C9 makes interplanar angles of 30.82 (7)° and 13.37 (7)° with the planes of 4-bromophenyl and 3,4,5-trimethoxyphenyl rings, respectively. The three methoxy groups of 3,4,5-trimethoxyphenyl unit have three difference orientations: one methoxy group (at atom C14 position) is co-planar with the attached benzene ring with torsion angle C18–O4–C14–C15 = 0.71 (17)° whereas the one at atom C12 position is twisted with the torsion angle C16–O2–C12–C11 = 10.38 (16)° and one is (+)-*syn*-clinally attached at atom C13 with the torsion angle C17–O3–C13—C14 = 74.48 (14)°. The bond distances are of normal values (Allen *et al.*, 1987) and are comparable with the closely related structures (Ng *et al.*, 2006; Patil *et al.*, 2006; 2007).

In the crystal packing (Fig. 2), the molecules are linked by weak C11—H11A···O1 intermolcular interactions (Table 1) into cyclic centrosymmetric *R*^2^_2_(14) dimers (Bernstein *et al.*, 1995). These dimers are stacked along the *c* axis (Fig. 2) and molecules within the stacks are interlinked by weak C17—H17C···O3 intermolecular interactions. The crystal is stabilized by weak C—H···O interactions (Table 1) and a C—H···π interaction (C16—H16B···*Cg*_1_ = 3.8080 (14) Å), where *Cg*_1_ is the centroid of the C10–C15 ring.

### Experimental

The title compound was synthesized by the condensation of 3,4,5-trimethoxybenzaldehyde (0.4 g, 2 mmol) with 4-bromoacetophenone (0.4 g, 2 mmol) in ethanol (50 ml) in the presence of 30% NaOH(aq) (10 ml). After stirring for 4 h, the resulting pale yellow solid appeared and was then collected by filtration, washed with distilled water, dried and purified by repeated recrystallization from acetone. Colorless block-shaped single crystals of the title compound suitable for *x*-ray structure determination were recrystalized from acetone/methanol (1:1 *v*/*v*) by the slow evaporation of the solvent at room temperature over several days, Mp. 403–404 K.

### Refinement

All H atoms were placed in calculated positions, with C—H = 0.93 Å, *U*_iso_ = 1.2*U*_eq_(C) for aromatic and CH and C—H = 0.96 Å, *U*_iso_ = 1.5*U*_eq_(C) for CH_3_ atoms. A rotating group model was used for the methyl groups. The highest residual electron density peak is located at 0.64 Å from C12 and the deepest hole is located at 0.24 Å from Br1.

### Crystal data

**Table:**

C_18_H_17_BrO_4_	*D*_x_ = 1.595 Mg m^−^^3^
*M**_r_* = 377.22	Melting point = 403–404 K
Tetragonal, *P*4_2_/*n*	Mo *K*α radiation, λ = 0.71073 Å
Hall symbol: -P 4bc	Cell parameters from 9693 reflections
*a* = 26.6517 (3) Å	θ = 1.1–40.0°
*c* = 4.4238 (1) Å	µ = 2.63 mm^−^^1^
*V* = 3142.28 (9) Å^3^	*T* = 100 K
*Z* = 8	Block, colorless
*F*(000) = 1536	0.55 × 0.12 × 0.12 mm

### Data collection

**Table:**

Bruker SMART APEXII CCD area-detector diffractometer	9693 independent reflections
Radiation source: fine-focus sealed tube	6638 reflections with *I* > 2σ(*I*)
graphite	*R*_int_ = 0.062
Detector resolution: 8.33 pixels mm^-1^	θ_max_ = 40.0°, θ_min_ = 1.1°
ω scans	*h* = −41→48
Absorption correction: multi-scan (*SADABS*; Bruker, 2005)	*k* = −48→44
*T*_min_ = 0.320, *T*_max_ = 0.726	*l* = −7→7
142737 measured reflections	

### Refinement

**Table:**

Refinement on *F*^2^	Primary atom site location: structure-invariant direct methods
Least-squares matrix: full	Secondary atom site location: difference Fourier map
*R*[*F*^2^ > 2σ(*F*^2^)] = 0.033	Hydrogen site location: inferred from neighbouring sites
*wR*(*F*^2^) = 0.097	H-atom parameters constrained
*S* = 1.07	*w* = 1/[σ^2^(*F*_o_^2^) + (0.0455*P*)^2^ + 0.774*P*] where *P* = (*F*_o_^2^ + 2*F*_c_^2^)/3
9693 reflections	(Δ/σ)_max_ = 0.003
211 parameters	Δρ_max_ = 0.71 e Å^−^^3^
0 restraints	Δρ_min_ = −0.56 e Å^−^^3^

### Special details

**Table:**

Experimental. The low-temperature data was collected with the Oxford Cryosystem Cobra low-temperature attachment.
Geometry. All e.s.d.'s (except the e.s.d. in the dihedral angle between two l.s. planes) are estimated using the full covariance matrix. The cell e.s.d.'s are taken into account individually in the estimation of e.s.d.'s in distances, angles and torsion angles; correlations between e.s.d.'s in cell parameters are only used when they are defined by crystal symmetry. An approximate (isotropic) treatment of cell e.s.d.'s is used for estimating e.s.d.'s involving l.s. planes.
Refinement. Refinement of *F*^2^ against ALL reflections. The weighted *R*-factor *wR* and goodness of fit *S* are based on *F*^2^, conventional *R*-factors *R* are based on *F*, with *F* set to zero for negative *F*^2^. The threshold expression of *F*^2^ > σ(*F*^2^) is used only for calculating *R*-factors(gt) *etc*. and is not relevant to the choice of reflections for refinement. *R*-factors based on *F*^2^ are statistically about twice as large as those based on *F*, and *R*- factors based on ALL data will be even larger. Data up to 2theta = 80 degrees is used in the final refinement

### Fractional atomic coordinates and isotropic or equivalent isotropic displacement parameters (Å^2^)

**Table:**

	*x*	*y*	*z*	*U*_iso_*/*U*_eq_	
Br1	0.332148 (4)	0.255150 (5)	−0.02867 (3)	0.01727 (4)	
O1	0.44846 (4)	0.45051 (4)	0.6855 (3)	0.02383 (19)	
O2	0.71139 (3)	0.49941 (3)	1.1449 (2)	0.01727 (16)	
O3	0.76246 (3)	0.42445 (3)	0.88670 (19)	0.01535 (15)	
O4	0.71518 (3)	0.34984 (3)	0.5922 (2)	0.01822 (16)	
C1	0.44558 (5)	0.32060 (5)	0.4779 (3)	0.0174 (2)	
H1A	0.4729	0.3097	0.5908	0.021*	
C2	0.41434 (4)	0.28571 (4)	0.3389 (3)	0.0167 (2)	
H2A	0.4201	0.2515	0.3625	0.020*	
C3	0.37450 (4)	0.30249 (4)	0.1647 (3)	0.01471 (19)	
C4	0.36484 (4)	0.35344 (4)	0.1273 (3)	0.01654 (19)	
H4A	0.3383	0.3642	0.0071	0.020*	
C5	0.39549 (4)	0.38788 (4)	0.2725 (3)	0.01630 (19)	
H5A	0.3890	0.4220	0.2523	0.020*	
C6	0.43604 (4)	0.37201 (5)	0.4488 (3)	0.01541 (19)	
C7	0.46729 (4)	0.41065 (5)	0.6074 (3)	0.0169 (2)	
C8	0.52093 (4)	0.39895 (5)	0.6544 (3)	0.0179 (2)	
H8A	0.5337	0.3689	0.5811	0.022*	
C9	0.55163 (4)	0.43071 (5)	0.8004 (3)	0.0167 (2)	
H9A	0.5369	0.4586	0.8901	0.020*	
C10	0.60602 (4)	0.42570 (4)	0.8320 (3)	0.01485 (19)	
C11	0.63128 (4)	0.46298 (4)	0.9952 (3)	0.01516 (19)	
H11A	0.6132	0.4878	1.0950	0.018*	
C12	0.68354 (4)	0.46278 (4)	1.0079 (2)	0.01394 (18)	
C13	0.71100 (4)	0.42475 (4)	0.8662 (3)	0.01360 (18)	
C14	0.68527 (4)	0.38623 (4)	0.7139 (3)	0.01435 (18)	
C15	0.63325 (4)	0.38689 (4)	0.6936 (3)	0.01565 (19)	
H15A	0.6165	0.3617	0.5887	0.019*	
C16	0.68428 (5)	0.53482 (5)	1.3242 (3)	0.0180 (2)	
H16A	0.7073	0.5587	1.4098	0.027*	
H16B	0.6670	0.5175	1.4837	0.027*	
H16C	0.6604	0.5521	1.1996	0.027*	
C17	0.78718 (5)	0.43783 (5)	0.6083 (3)	0.0191 (2)	
H17A	0.8228	0.4337	0.6311	0.029*	
H17B	0.7799	0.4722	0.5601	0.029*	
H17C	0.7754	0.4165	0.4485	0.029*	
C18	0.69070 (5)	0.31032 (5)	0.4311 (3)	0.0198 (2)	
H18A	0.7153	0.2866	0.3617	0.030*	
H18B	0.6731	0.3241	0.2608	0.030*	
H18C	0.6673	0.2938	0.5625	0.030*	

### Atomic displacement parameters (Å^2^)

**Table:**

	*U*^11^	*U*^22^	*U*^33^	*U*^12^	*U*^13^	*U*^23^
Br1	0.01487 (6)	0.01441 (6)	0.02252 (6)	−0.00131 (4)	−0.00146 (4)	−0.00109 (4)
O1	0.0156 (4)	0.0196 (4)	0.0364 (5)	0.0027 (3)	−0.0021 (4)	−0.0084 (4)
O2	0.0142 (4)	0.0161 (4)	0.0215 (4)	−0.0018 (3)	−0.0011 (3)	−0.0040 (3)
O3	0.0099 (3)	0.0223 (4)	0.0138 (3)	0.0010 (3)	−0.0009 (3)	0.0006 (3)
O4	0.0127 (4)	0.0169 (4)	0.0250 (4)	0.0019 (3)	−0.0016 (3)	−0.0061 (3)
C1	0.0148 (5)	0.0171 (5)	0.0204 (5)	0.0026 (4)	−0.0025 (4)	−0.0007 (4)
C2	0.0168 (5)	0.0140 (5)	0.0193 (5)	0.0026 (4)	−0.0012 (4)	0.0000 (4)
C3	0.0123 (4)	0.0145 (5)	0.0174 (5)	−0.0013 (3)	0.0011 (4)	−0.0005 (4)
C4	0.0132 (5)	0.0153 (5)	0.0211 (5)	0.0012 (4)	−0.0019 (4)	0.0017 (4)
C5	0.0131 (5)	0.0137 (5)	0.0221 (5)	0.0012 (4)	−0.0009 (4)	0.0008 (4)
C6	0.0115 (4)	0.0165 (5)	0.0182 (5)	0.0005 (4)	0.0003 (4)	−0.0012 (4)
C7	0.0120 (5)	0.0179 (5)	0.0209 (5)	0.0003 (4)	−0.0006 (4)	−0.0018 (4)
C8	0.0121 (5)	0.0186 (5)	0.0231 (5)	0.0014 (4)	−0.0007 (4)	−0.0040 (4)
C9	0.0122 (5)	0.0158 (5)	0.0220 (5)	0.0004 (4)	0.0002 (4)	−0.0014 (4)
C10	0.0115 (4)	0.0145 (5)	0.0185 (5)	−0.0001 (3)	−0.0003 (3)	0.0001 (4)
C11	0.0124 (4)	0.0141 (5)	0.0190 (5)	−0.0002 (3)	0.0000 (4)	0.0001 (4)
C12	0.0130 (4)	0.0133 (4)	0.0156 (4)	−0.0011 (3)	−0.0005 (3)	0.0007 (3)
C13	0.0110 (4)	0.0156 (5)	0.0143 (4)	0.0000 (3)	−0.0008 (3)	0.0007 (3)
C14	0.0130 (4)	0.0137 (4)	0.0163 (4)	0.0014 (3)	−0.0008 (3)	−0.0003 (4)
C15	0.0131 (5)	0.0147 (5)	0.0191 (5)	0.0000 (4)	−0.0013 (4)	−0.0015 (4)
C16	0.0196 (5)	0.0159 (5)	0.0186 (5)	0.0003 (4)	−0.0014 (4)	−0.0019 (4)
C17	0.0148 (5)	0.0258 (6)	0.0168 (5)	−0.0009 (4)	0.0009 (4)	0.0024 (4)
C18	0.0170 (5)	0.0165 (5)	0.0259 (6)	0.0002 (4)	−0.0004 (4)	−0.0051 (4)

### Geometric parameters (Å, °)

**Table:**

Br1—C3	1.8967 (11)	C8—H8A	0.9300
O1—C7	1.2247 (15)	C9—C10	1.4624 (16)
O2—C12	1.3678 (14)	C9—H9A	0.9300
O2—C16	1.4292 (15)	C10—C11	1.4007 (16)
O3—C13	1.3746 (13)	C10—C15	1.4039 (16)
O3—C17	1.4413 (15)	C11—C12	1.3941 (16)
O4—C14	1.3658 (14)	C11—H11A	0.9300
O4—C18	1.4294 (15)	C12—C13	1.3984 (16)
C1—C2	1.3916 (17)	C13—C14	1.4065 (16)
C1—C6	1.3997 (17)	C14—C15	1.3894 (16)
C1—H1A	0.9300	C15—H15A	0.9300
C2—C3	1.3862 (16)	C16—H16A	0.9600
C2—H2A	0.9300	C16—H16B	0.9600
C3—C4	1.3917 (16)	C16—H16C	0.9600
C4—C5	1.3866 (17)	C17—H17A	0.9600
C4—H4A	0.9300	C17—H17B	0.9600
C5—C6	1.3981 (16)	C17—H17C	0.9600
C5—H5A	0.9300	C18—H18A	0.9600
C6—C7	1.4990 (17)	C18—H18B	0.9600
C7—C8	1.4777 (16)	C18—H18C	0.9600
C8—C9	1.3428 (17)		
			
C12—O2—C16	116.30 (9)	C12—C11—C10	119.88 (11)
C13—O3—C17	113.47 (9)	C12—C11—H11A	120.1
C14—O4—C18	116.97 (9)	C10—C11—H11A	120.1
C2—C1—C6	120.31 (11)	O2—C12—C11	123.86 (11)
C2—C1—H1A	119.8	O2—C12—C13	115.59 (10)
C6—C1—H1A	119.8	C11—C12—C13	120.51 (10)
C3—C2—C1	119.24 (11)	O3—C13—C12	119.79 (10)
C3—C2—H2A	120.4	O3—C13—C14	120.92 (10)
C1—C2—H2A	120.4	C12—C13—C14	119.25 (10)
C2—C3—C4	121.50 (11)	O4—C14—C15	124.47 (10)
C2—C3—Br1	119.46 (9)	O4—C14—C13	115.00 (10)
C4—C3—Br1	119.04 (9)	C15—C14—C13	120.54 (10)
C5—C4—C3	118.81 (11)	C14—C15—C10	119.81 (11)
C5—C4—H4A	120.6	C14—C15—H15A	120.1
C3—C4—H4A	120.6	C10—C15—H15A	120.1
C4—C5—C6	120.89 (11)	O2—C16—H16A	109.5
C4—C5—H5A	119.6	O2—C16—H16B	109.5
C6—C5—H5A	119.6	H16A—C16—H16B	109.5
C5—C6—C1	119.21 (11)	O2—C16—H16C	109.5
C5—C6—C7	118.87 (11)	H16A—C16—H16C	109.5
C1—C6—C7	121.89 (11)	H16B—C16—H16C	109.5
O1—C7—C8	122.66 (11)	O3—C17—H17A	109.5
O1—C7—C6	120.02 (11)	O3—C17—H17B	109.5
C8—C7—C6	117.29 (10)	H17A—C17—H17B	109.5
C9—C8—C7	121.62 (11)	O3—C17—H17C	109.5
C9—C8—H8A	119.2	H17A—C17—H17C	109.5
C7—C8—H8A	119.2	H17B—C17—H17C	109.5
C8—C9—C10	126.33 (11)	O4—C18—H18A	109.5
C8—C9—H9A	116.8	O4—C18—H18B	109.5
C10—C9—H9A	116.8	H18A—C18—H18B	109.5
C11—C10—C15	119.93 (10)	O4—C18—H18C	109.5
C11—C10—C9	117.44 (10)	H18A—C18—H18C	109.5
C15—C10—C9	122.55 (10)	H18B—C18—H18C	109.5
			
C6—C1—C2—C3	1.71 (18)	C16—O2—C12—C11	10.38 (16)
C1—C2—C3—C4	−0.42 (18)	C16—O2—C12—C13	−171.99 (10)
C1—C2—C3—Br1	179.40 (9)	C10—C11—C12—O2	175.59 (11)
C2—C3—C4—C5	−1.05 (18)	C10—C11—C12—C13	−1.93 (17)
Br1—C3—C4—C5	179.12 (9)	C17—O3—C13—C12	−107.90 (12)
C3—C4—C5—C6	1.25 (18)	C17—O3—C13—C14	74.48 (14)
C4—C5—C6—C1	0.01 (18)	O2—C12—C13—O3	3.79 (15)
C4—C5—C6—C7	−178.49 (11)	C11—C12—C13—O3	−178.50 (10)
C2—C1—C6—C5	−1.51 (18)	O2—C12—C13—C14	−178.55 (10)
C2—C1—C6—C7	176.94 (11)	C11—C12—C13—C14	−0.84 (17)
C5—C6—C7—O1	28.97 (18)	C18—O4—C14—C15	0.71 (17)
C1—C6—C7—O1	−149.50 (13)	C18—O4—C14—C13	−179.04 (11)
C5—C6—C7—C8	−149.21 (12)	O3—C13—C14—O4	−0.08 (16)
C1—C6—C7—C8	32.33 (17)	C12—C13—C14—O4	−177.72 (10)
O1—C7—C8—C9	3.4 (2)	O3—C13—C14—C15	−179.84 (10)
C6—C7—C8—C9	−178.44 (12)	C12—C13—C14—C15	2.53 (17)
C7—C8—C9—C10	−173.04 (12)	O4—C14—C15—C10	178.83 (11)
C8—C9—C10—C11	−179.68 (12)	C13—C14—C15—C10	−1.43 (18)
C8—C9—C10—C15	3.6 (2)	C11—C10—C15—C14	−1.35 (18)
C15—C10—C11—C12	3.03 (17)	C9—C10—C15—C14	175.30 (11)
C9—C10—C11—C12	−173.80 (11)		

### Hydrogen-bond geometry (Å, °)

**Table:**

*D*—H···*A*	*D*—H	H···*A*	*D*···*A*	*D*—H···*A*
C11—H11A···O1^i^	0.93	2.52	3.4391 (16)	170
C17—H17C···O3^ii^	0.96	2.52	3.2789 (16)	136
C16—H16B···Cg1^iii^	0.96	2.97	3.8080 (14)	147

Symmetry codes: (i) −*x*+1, −*y*+1, −*z*+2; (ii) *x*, *y*, *z*−1; (iii) *x*, *y*, *z*+1.
